# Brief intervention for agrammatism in Primary Progressive Nonfluent Aphasia: A case report

**DOI:** 10.1590/S1980-57642014DN83000014

**Published:** 2014

**Authors:** Thais Helena Machado, Aline Carvalho Campanha, Paulo Caramelli, Maria Teresa Carthery-Goulart

**Affiliations:** 1 1Grupo de Neurologia Cognitiva e do Comportamento, Departamento de Clínica Médica, Faculdade de Medicina da Universidade Federal de Minas Gerais, Belo Horizonte MG, Brazil.; 2 Núcleo de Cognição e Sistemas Complexos e Centro de Matemática, Computação e Cognição da Universidade Federal do ABC, Santo André SP, Brazil. Grupo de Neurologia Cognitiva e do Comportamento, Departamento de Neurologia, Hospital das Clínicas da Faculdade de Medicina da Universidade de São Paulo, São Paulo SP, Brazil.

**Keywords:** primary progressive aphasia, treatment, speech and language therapy, intervention, cognitive rehabilitation

## Abstract

The non-fluent and agrammatic variant of Primary Progressive Aphasia (NFPPA) is characterized by reduced verbal production with deficits in building grammatically correct sentences, involving dysfunctions in syntactic and morphological levels of language. There are a growing number of studies about non-pharmacological alternatives focusing on the rehabilitation of functional aspects or specific cognitive impairments of each variant of PPA. This study reports a short-term treatment administered to a patient with NFPPA focusing on the production of sentences. The patient had significant reduction in verbal fluency, use of keywords, phrasal and grammatical simplifying as well as anomia. Using the method of errorless learning, six sessions were structured to stimulate the formation of sentences in the present and past with the cloze technique. The patient had improvement restricted to the strategy, with 100% accuracy on the trained phrases and generalization to untrained similar syntactic structure after training. These results persisted one month after the treatment.

## Abstract (pt)

A variante não fluente e agramática da Afasia Progressiva Primária (APPNF) caracteriza-se por redução da produção verbal com falhas para construir sentenças gramaticalmente corretas, envolvendo disfunções nos níveis sintático e morfológico da linguagem. É crescente o número de pesquisas sobre alternativas não farmacológicas enfocando a reabilitação de aspectos funcionais ou comprometimentos cognitivos específicos de cada variante das APP. Relatar um tratamento de curta-duração administrado a um paciente com APPNF enfocando a produção de sentenças. O paciente apresentava redução importante da fluência verbal, uso de palavras-chave, simplificação frasal e gramatical, além de anomia. Utilizando o método de aprendizado sem erros, foram estruturadas seis sessões para estimular a formação de frases no presente e no passado com a técnica de *cloze*. O paciente apresentou melhora restrita à estratégia, com 100% de acerto nas frases treinadas e generalização para as não-treinadas de estrutura sintática semelhante após o treinamento. Os resultados foram mantidos um mês após o tratamento.

## INTRODUCTION

Syndromes in which there is progressive and relatively isolated language impairment without other significant cognitive deficits are known as Primary Progressive Aphasia (PPA). Patients with PPA present preservation of function in activities that do not depend on the use of language.

Clinical cases with slowly progressive aphasia were described by Mesulam.^1^ However, only recently consensual diagnostic criteria were proposed by a committee of experts, thus allowing better comparisons across studies.^2^ The researchers have proposed inclusion and exclusion criteria for PPA and classification into three different variants: nonfluent / agrammatic (NFPPA), semantic (PPAS) and logopenic (PPAL). The main symptom of the nonfluent variant is agrammatism and/or apraxia of speech. The agrammatic subtype in particular is characterized by reduced verbal output and impairment in the production of grammatically correct sentences, involving disorders in syntactic and morphological levels of language. The comprehension of complex sentences can also be altered. However, simple sentences and words (concepts) are well understood.^3,4^

Effective pharmacological treatments for PPA are not currently available,^5-8^ which has prompted research into non-pharmacological alternatives (behavioral) focusing on functional aspects or specific cognitive impairments of each variant. Nonpharmacological interventions are "any theoretically based, nonchemical, focused and replicable intervention, conducted with the patient or the caregiver, which may potentially provide some relevant benefit".^9^

Carthery-Goulart et al.,^10^ in a systematic review of the literature about non-pharmacological interventions in PPA, established evidence-based recommendations for the clinical practice of cognitive rehabilitation in this population. The authors found that most studies reviewed involved interventions directed to specific cognitive deficits (especially anomia) and consisted of case studies (single or multiple) with appropriate methodology to assess therapeutic efficacy, comprising different item groups (trained and untrained), multiple baseline evaluation design and investigation of outcomes immediately after treatment and at different periods posttreatment (follow-up). By the number of studies with adequate experimental design, the authors concluded that interventions focusing on the naming deficit for the semantic variant of PPA can be recommended with level of evidence III. However, for all other variants and intervention techniques there are few reports and more studies on interventions that may benefit these patients are needed. Among the 39 studies analyzed, only 20% of them included NFPPA patients. Of these, only one case report focused on the treatment of agrammatism.^11^

In this context, the aim of the present study was to report the short-term effects of speech therapy intervention in a patient with NFPPA, focusing on the production of sentences.

According to the sentence production model proposed by Bock and Levelt,^12^ agrammatism may be due to problems in the functional and/or positional levels of grammatical encoding. Problems affecting the functional level impair lexical selection and assignment of thematic roles to the verb. Problems in the positional level affect the assembly of words in a sentence frame and the generation of appropriate inflections. Therapy directed to agrammatism may focus on each of these levels, according to the clinical profile of the patient.

In the current study, an extensive language assessment was conducted and the grammatical encoding dysfunction interpreted based on Bock and Levelt's framework. The implementation of the treatment, along with the immediate results post-treatment and after one month of follow-up are described.

## METHOD

This study is part of a Brazilian multicentre initiative of validation of intervention techniques directed to specific impairments in PPA patients. Our main goal was to gather evidence-based language intervention techniques which can be implemented in public health services, characterized by intensive and short-term treatment phases, with periodic follow-up and maintenance cycles. We have been developing semi-structured programs for each type of deficit (i.e. anomia, agrammatism) whose items can be individualized and adjusted for severity in each case. The strategies are based on Cognitive Neuropsychology and Psycho-linguistics models. The project and the free and informed consent form were approved by the Federal University of Minas Gerais Research Ethics Committee (protocol 666.666).

**Subject.** Case-report: EEP, a male right-handed patient aged 66 years with 15 years of schooling is reported. On initial assessment he reported progressive word-finding difficulties which had started three years earlier. He was still able to drive and manage his own company, reporting no other impairment. The patient was referred for cognitive rehabilitation after receiving a diagnosis of NFPPA following extensive investigation by the multidisciplinary team at the Cognitive and Behavioral Neurology Clinics of the UFMG. He presented with an isolated language deficit with relative preservation of other cognitive functions and independence in activities of daily living except for those requiring language abilities. Regarding his cognitive profile, EPP scored 27 on the Mini-Mental State Exam and 12 on the Frontal Assessment Battery (maximum 18), indicating a mild impairment of executive function.

## Intervention description

*Phase 1: Language assessment and decision about the focus and start point of the intervention* - In functional communicative situations, EEP demonstrated preservation of comprehension skills, suitable communicative exchange shifts and other pragmatic resources but exhibited marked expression deficits with anomia and word-finding difficulties, significant output reduction using only content words (especially nouns) and simplified phrases and grammar (omission of function words and inflections). In addition to the linguistic changes, the patient also manifested mild apraxia of speech. The results of formal language tests (Table 1) were suggestive of anomia, with benefit from phonemic cues (lexical access deficit), reduced category and letter verbal fluency, and sentence comprehension deficits, as evaluated by the Token Test^13^ and the Test for Reception of Grammar (TROG-2).^14,15^ The Token Test evaluates comprehension of commands and is useful when trying to understand the influence of extension and syntax complexity on comprehension. Parts 1, 2 and 3 check word knowledge and syntactically simple/short commands. Part 4 tests long but simple constructions, whereas Part 5 tests syntactically complex sentences. The results of EEP indicated problems both related to phonological working memory (Part 4) and syntactic comprehension deficits (Part 5). To better characterize the syntactic deficit, EEP was assessed with TROG. This test contains 80 items, divided into 20 blocks, each testing a different lexical, morphosyntactic or syntactic structure. Each item is read aloud to the subject who has to choose from four drawings the one which best depicts the item. A block is scored as passed only if all 4 items within it are answered correctly. In addition, a final score is obtained by summing all the correct responses. EEP passed only 6/20 blocks. An analysis of error patterns, revealed four failed blocks due to attentional problems (only 1 error in the block) and genuine morphosyntactic deficits in 10 blocks (2 or more errors in blocks C,E,K,L,M,N,P,R,S,T). EEP presented difficulties with all reversible constructions, zero anaphora, pronouns (gender, number and binding), singular/plural inflections, relative clauses and centre embedded sentences). In speech production from visual stimuli (Cookie theft)^16^ a very low occurrence of verbs (only 3: to leak, fall and be) was observed and errors in verb inflections. In reading, the patient showed omission and transposition of phonemes and omitted articles and conjunctions, but with satisfactory comprehension. In writing, EEP committed only simple spelling errors.

**Table 1:** Performance on language tasks before intervention.

**Grammatical comprehension**	TROG^14,15^	Total score 53/80
		Passed Blocks 6/20
	Token test^13^	1st part: 100%
		2nd part: 100%
		3rd part: 70%
		4th part: 10%
		5th part: 23.80%
**Naming**	Boston Naming Test^16^	35/60 (improvement with phonemic cues)
**Verbal fluency**	Animals^17^	5
	FAS^18^	2
**Oral narration**	Cookie theft (Boston test)^16^	mulher homem pote... mulher prato pia vazando... caido caido banco é... prato xícara... num sei o que não... janela cortina pia... água... planta « woman man pot... woman dish sink leaking... fallen fallen stock is... dish cup... I don’t know what ... window curtain sink... water plant »

We applied a sentence completion task and verified that although the patient was unable to produce complete sentences spontaneously or in picture description, he was able to read and repeat short sentences of simple grammatical structure (subject-verb-complement). In a cloze task (a written sentence was presented with a gap in the position of the verb) with these types of sentences, when given the verb in the infinitive form, he was able to use it in the sentence. However, he occasionally presented morphological errors, either omitting or substituting inflections both referring to subject pronouns and tense. By analysing comprehension and production tasks, we interpreted the grammatical encoding deficit as due to problems in understanding thematic roles, characterized by difficulties with reversible sentences and omission of verbs and function words (lexical access) and also problems assembling words in a sentence frame and generating appropriate inflections, characterized by inability to produce complex sentences and verb inflections. Therefore, the deficits affected both functional and positional levels in Bock and Levelt's framework. Our goal was to focus first on the positional level, by practicing present and past verb inflections and production of "subject-verb-complement" sentence structures.

*Phase 2: Implementation of the treatment* - Six rehabilitation sessions (twice a week, lasting for 30 minutes each, with daily workouts at home) were carried out for three weeks. The purpose was to improve his syntactic skills, more specifically, verb inflections, for construction of coherent simple sentences, since the patient used nouns and simple phrases to communicate.

For regular practice, 20 high-frequency regular verbs were selected (transitive direct and indirect). The selection of verbs was based on common daily activities (examples: to run, eat, cook, shop, work) and verb frequency was confirmed at the *corpus* AC/DC (http://www.linguateca.pt/ACDC/). A set of 40 sentences was built to be completed with the same 20 verbs in the present and in the past (an adverb at the beginning of the sentence cued the verb tense, i.e. "Every day"; "Yesterday"). In all sessions the patient was asked to complete the gaps in all 40 sentences, always presented in the same order, using a verb that was given in the infinitive form by the therapist.

Another set of 40 sentences with different regular verbs were selected, but was not trained, to serve as comparison and for analysis of generalization.

*Phase 3: Intervention strategy* - The first goal was to achieve accurate production of Subject-Verb-Object sentences. For this, in the first session an explanation of the rules of conjugation of regular verbs in the two different tenses was given. Subsequently, a written cue (verb in the infinitive) was provided by the therapist, to be used in the completion of a given written sentence. In this situation, the baseline assessment showed 85% correct responses. For treatment, the therapist offered the model aloud (verb inflection) and the patient was asked to read the sentence and reproduce the verb form in the correct position in the sentence (blank). The sentence was then partially covered and the patient was asked to say the full sentence aloud again. When necessary, a picture was presented as support. According to the technique of errorless learning, models and cue were gradually removed until the patient was able to complete the sentence and say it aloud without any cues. The sentences were trained in the present and past tenses.

## RESULTS

Table 2 shows the results of trained and untrained items pre- and post-intervention. Pictures were removed from training in the second session. In the fourth session both models of the inflected verb and the verb in the infinitive were no longer needed for cues. The Wilcoxon signed ranks test was used to compare trained items (pre and post-treatment) and untrained items (pre and post-treatment). A significant increase in correct sentences in the post-treatment was evident both for trained (p<0.05) and untrained items (p<0.01). It is important to highlight that pre-treatment measures refer to patient production with a verb in the infinitive offered as a cue whereas at post-treatment and follow-up no cue was needed.

**Table 2:** Results of trained and untrained sentences pre- and postintervention

**Results of trained sentences pre- and post-intervention**	
Pre	Post
85%	100% p<0.05
**Results of untrained sentences pre- and post-intervention**	
Pre	Post
70%	100% p<0.01

The narrative in the oral description of the "Cookie theft"^15^ figure after the intervention was: *o menino caiu do banquinho... a menina... a menina... caiu do banquinho do biscoito... ela lavou os pratos e a torneira derrama... a cortina está aberta... os armários estão fechados.* "The boy fell off the stool... the girl... the girl... fell off the stool of the cookie... she washed the dishes and pour the tap... the curtain is opened... the cabinets are closed"

During the month following the end of training, the patient underwent rehabilitation directed only to apraxia of speech. One month after the end of language training the patient was reevaluated and maintained 100% accuracy in both trained and untrained phrases. On the same day the patient was submitted to grammatical comprehension tests. He was able to score one point on the TROG^14,15^ (53 pre- and 54 post-intervention), but presented greater difficulty on the Token test^13^ (100%, 83.33%, 30%, 10% and 14.28% on each part, respectively), showing increased deficits in attention and phonological working memory.

## DISCUSSION

Non-pharmacological interventions have been proposed as promising alternatives for the treatment of cognitive deficits following PPA (for a review see Carthery-Goulart et al.)^10^. Most studies have focused on the treatment of lexical or semantic deficits since these are common features of the three most common variants of PPA and a frequent concern of patients. The current study describes the short-term effects of an intervention program designed for a patient with nonfluent PPA focusing on sentence production. Although agrammatism is a core feature of NFPPA, to our knowledge there is only one case-study describing strategies that can be applied to remediate this deficit in progressive aphasia.^11^

Agrammatism is characterized by three main features: omission or substitution of morphology; reduced variety of grammatical forms (constructional deficit, characterized by typically producing declarative sentences with canonical word order); and slow rate of speech, even in patients without articulatory problems.^19^ These deficits are usually explained in terms of impairment of morphosyntactic knowledge or specific restriction of grammatical processing skills.^19^ The deficits presented by the patient described, and possibly by others in the initial stages of NFPPA, seem to be better explained by the second description. According to Kolk,^19^ agrammatic patients adapt the syntactic complexity of their utterances to their limited processing capacity, which is supported by the observation of variance of performance among tasks. The patient's output was worse in picture description compared to sentence completion/cloze, which are more constrained tasks. In fact, he was able to build more accurate sentences in cued tasks, which suggests that morphosyntactic knowledge was more preserved than access to it.

The treatment proposed for EEP increased awareness of sentence structure and its components (meta-linguistic skills) and at the same time provided intensive training on building simple canonical sentences. We used principles of errorless learning by offering cues (verb in the infinitive) and asking for repetition of tense inflections. In this sense, the treatment exploited the remaining capacity both of declarative and non-declarative memory systems. The patient was able to learn the strategy and to generalize it to trained and untrained sentences of similar grammatical structure. He was also able to use the strategy in picture description. However, generalization was not observed in sentence comprehension. As our treatment did not address different (and more complex) sentence structures we did not expect this generalization to occur. In fact, what we observed was the generalization of the trained strategy.

Our findings are comparable to those of Schneider et al.,^11^ although these researchers employed a different type of intervention. In their study, treatment was aimed at cueing verb tense inflections by the use of gestures. The investigators observed improved production of sentences on trained items and untrained verbs within trained tenses. In our study, we believe that treatment was successful by reducing adaptive behavior (or "maladaptive", because the patient was functioning below his capabilities). In neurodegenerative diseases, avoiding maladaptation and maintaining an optimum level of functioning must also be a goal when trying to delay deterioration of language function.

Although verb retrieval was not tested before and after treatment, we believe that the strategy of intervention might also have contributed to facilitate verb retrieval, because after treatment the patient was able to work on the cloze task without being given the target verb. Improving verb retrieval has been shown to be a successful strategy for improving sentence production in chronic aphasia^20^ and this hypothesis must be addressed in future studies.

After the period of syntactic training, a treatment focused on motor programming (apraxia of speech) for a month was chosen, since it is a distinct task and was not expected to have had an impact on syntactic skills up to the re-evaluation.

Some limitations of the current work is that samples of spontaneous speech were not recorded at baseline to be compared with the levels obtained post-treatment. Therefore, our conclusions are based solely on observations of the improved performance on picture description (which is also a constrained task) and in the generalization of the strategy to untrained sentences of similar structures but not to comprehension tasks. We also recognize that the number of items (trained and untrained) needs to be increased in future studies. Moreover, a verb naming task would have helped in the interpretation of gains in terms of improvement of verb retrieval or sentence structure awareness. For training, a fixed order of sentence presentation was used, however this seemed not to affect the results of the treatment, since learning was generalized to untrained sentences and to different tasks (picture description).

We aim to address these hypotheses in future studies. As a continuation of this work, in the next cycle of intervention with this patient, we intend to add more complex sentence structures and address syntactic comprehension.

The results of this study lend support for the use of linguistic-based treatment strategies in PPA. Further studies in PPA are needed to gather evidence-based treatment options to assist in clinical practice.
